# How Does Deep Brain Stimulation Change the Course of Parkinson's Disease?

**DOI:** 10.1002/mds.29052

**Published:** 2022-05-12

**Authors:** Philipp Mahlknecht, Thomas Foltynie, Patricia Limousin, Werner Poewe

**Affiliations:** ^1^ Department of Neurology Innsbruck Medical University Innsbruck Austria; ^2^ Department of Clinical and Movement Neurosciences UCL Institute of Neurology, National Hospital for Neurology and Neurosurgery London United Kingdom

**Keywords:** nursing home placement, mortality, disease‐modifying, disease‐modification, neuroprotection

## Abstract

A robust body of evidence from randomized controlled trials has established the efficacy of deep brain stimulation (DBS) in reducing *off* time and dyskinesias in levodopa‐treated patients with Parkinson's disease (PD). These effects go along with improvements in *on* period motor function, activities of daily living, and quality of life. In addition, subthalamic DBS is effective in controlling drug‐refractory PD tremor. Here, we review the available data from long‐term observational and controlled follow‐up studies in DBS‐treated patients to re‐examine the persistence of motor and quality of life benefits and evaluate the effects on disease progression, major disability milestones, and survival. Although there is consistent evidence from observational follow‐up studies in DBS‐treated patients over 5–10 years and beyond showing sustained improvement of motor control, the long‐term impact of DBS on overall progression of disability in PD is less clear. Whether DBS reduces or delays the development of later motor and non‐motor disability milestones in comparison to best medical management strategies is difficult to answer by uncontrolled observational follow‐up, but there are signals from controlled long‐term observational studies suggesting that subthalamic DBS may delay some of the late‐stage disability milestones including psychosis, falls, and institutionalization, and also slightly prolongs survival compared with matched medically managed patients. These observations could be attributable to the sustained improvements in motor function and reduction in medication‐induced side effects, whereas there is no clinical evidence of direct effects of DBS on the underlying disease progression. © 2022 The Authors. *Movement Disorders* published by Wiley Periodicals LLC on behalf of International Parkinson and Movement Disorder Society

Parkinson's disease (PD) stands out among the neurodegenerative diseases by the availability of powerful symptomatic therapies.^1^, ^2^ Treatment with levodopa has transformed the lives of millions of people with PD since it was first introduced more than 50 years ago and has remained the gold standard of symptomatic antiparkinsonian drug efficacy. However, levodopa has also brought forth a new source of disability by inducing motor complications such as response fluctuations and dyskinesias in the majority of patients after years of treatment.^1^ In addition, levodopa and other available PD therapies do not seem to prevent or slow the underlying progression of the disease.^3^, ^4^ The late stages of PD are characterized by poorly levodopa‐responsive gait and balance difficulties, dysarthria, and dysphagia together with non‐motor symptoms such as orthostatic hypotension, depression, cognitive decline, dementia, and psychosis that can lead to severe disability and requirement of nursing home care.^5^, ^6^, ^7^

The introduction of deep brain stimulation (DBS) targeting the subthalamic nucleus (STN) or the globus pallidus internus (GPi) almost 30 years ago^8^ has changed the outlook for patients with pharmacologically uncontrollable motor fluctuations and levodopa‐induced dyskinesias and those suffering from drug‐refractory PD tremor. Superiority of DBS over best medical treatment (BMT) has since been established in several carefully conducted, properly powered, randomized controlled trials (RTCs) (Table 1).^9^, ^10^, ^11^, ^12^, ^13^, ^14^ These trials consistently showed marked reductions in *off* medication motor severity (part three of the Unified PD Rating Scale [UPDRS‐III]) of 30%–50% and increase in daily *on* time of 2–5 hours, along with improvements of dyskinesias, activities of daily living (ADL; as per UPDRS‐II), and quality of life (QoL) in comparison to BMT. Improvements in QoL were most profound in the mobility, ADLs, and bodily discomfort domains^9^, ^10^, ^14^ and seem to be related to improvements in *off* motor function and reduction in daily *off* time.^15^ All trials used the STN as a target, except for one, which also used the GPi and showed similar efficacy of either STN‐DBS or GPi‐DBS versus BMT.^12^ Two additional trials compared GPi‐DBS and STN‐DBS without a BMT arm and found no differences in changes of primary outcome measures.^16^, ^17^ However, both showed greater levodopa equivalent dose reductions and one showed greater improvements in *off* medication motor functioning with STN‐DBS,^16^ findings that were confirmed by a recent large meta‐analysis.^18^ Based on this solid evidence STN‐DBS and GPi‐DBS are firmly established treatment modalities to improve motor fluctuations and dyskinesias in advanced PD.^19^ STN‐DBS is also highly efficacious in reducing or even abolishing drug refractory PD tremor and is widely used in clinical practice for this indication. In fact, the only RCT reporting changes in individual motor signs found tremor to be best responsive to STN‐DBS, followed by rigidity, gait, and bradykinesia.^13^

**TABLE 1:** Randomized controlled trials of DBS and BMT versus BMT alone

	Deuschl et al^9^	Williams et al^10^ (PD SURG)	Weaver et al^12^	Okun et al^11^ (SJM DBS)	Schuepbach et al^14^ (EARLY‐STIM)	Vitek et al^13^ (INTREPID)	Mean
N (DBS/BMT)	78/78	162/153	121/134^a^	101/35	120/123	121/39	
Study duration (month)	6	12	6	3	24	3	
Baseline characteristics							
Mean age (y)	61	59	62	60	52^b^	60	
Mean disease/treatment duration (y)	13	11	12	12	7.5	10	
Mean fluctuation duration (y)	n.g.	n.g.	n.g.	n.g.	1.5 (≤3)^b^	n.g.	
Median H&Y (*off* med)	~4	~3	~3	~3	<3^b^	n.g.	
Outcomes							
UPDRS III (*off* meds/ON stim), %	39.3^c^	33.0	24.9	30.8	49.1	28.7	34.3
UPDRS II (*off* meds/ON stim), %	43.7	22.8	24.1	n.g.	41.5	n.g.	33.0
UPDRS IV (*on* meds/ON stim), %	n.g.	48.9	31.6	32.2	73.4	n.g.	49.1
*Off* time reduction, %	67.7 (4.2 h)	n.g.	44.2 (2.4 h)	n.g.	45.4 (1.8 h)	n.g.	52.4 (2.8 h)
*On* time increase (without troublesome dyskinesias), %	154.2 (4.9 h)	n.g.	67.2 (4.6 h)^c^	35.7 (2.5 h)^c^	18.4 (1.9 h)	47.9 (3.0 h)^c^	64.7 (3.4 h)
PDQ‐39, %	25.4^c^	11.8^c^	19.4	n.g.	25.5^c^	38.2	23.9
LEDD reduction, %	39.4	34	24.2	21.3	65.9	n.g.	37.0

Changes in outcome measures are given in percent (%) improvement from baseline with neurostimulation in relation to best medical treatment. All studies used the nucleus subthalamicus (STN) as stimulation target, except for one study using either pallidal or subthalamic stimulation (Weaver et al).

^a^ Randomized to either STN (n = 60) or GPi (n = 61) within the DBS group.

^b^ In the EARLYSTIM trial age 18–60 y, a motor fluctuation duration of ≤3 y, and H&Y <3 *on*‐medication were inclusion criteria.

^c^ Indicates primary outcomes of respective studies.

Abbreviations: BMT, best medical treatment; DBS, deep brain stimulation; H&Y, Hoehn and Yahr Scale score; h, hour; n.g., not given; PDQ‐39, Parkinson's disease questionnaire 39, UPDRS, Unified PD rating scale.

Although most trials included patients with a mean age of around 60 years, mean disease durations of 10–13 years, and a long‐standing history of motor complications, the EARLYSTIM trial has shown similar benefits of subthalamic neurostimulation in younger patients with much shorter disease duration and motor complication history (Table 1).^14^ However, it remains uncertain if and to what extent DBS, especially if introduced early in the course of the disease, alters the clinical progression and long‐term outcome of PD. This issue is also of relevance in view of experimental animal studies suggesting “neuroprotective” effects of STN‐DBS.^20^, ^21^

Hence, we aimed to tackle this question by comprehensively reviewing available data from long‐term follow‐up studies over at least 5 years in terms of DBS impact on motor functioning, QoL, as well as disease progression with a specific focus on major disability milestones, need for institutional care, and survival. Of note, the vast majority of published long‐term studies included patients with subthalamic neurostimulation, such that the present review focuses on STN‐DBS, but also refers to the few relevant studies in patients with GPi‐DBS.

## Long‐Term Impact of DBS on Motor Symptoms and Quality of Life

Although study durations of RCTs do not allow for conclusions on the long‐term persistence of observed benefits, open label follow‐up studies of two RCTs comparing STN‐DBS with GPi‐DBS^16^, ^17^ have shown sustained improvement of motor features (fluctuations, dyskinesias, *on*‐ and *off*‐medication motor function) and ADL‐scores at 36 months with both stimulation targets,^22^, ^23^ as well as sustained reductions in total dopaminergic drug dose in those treated with STN‐DBS.^23^ Ten‐year follow‐up data of one of these trials, published in abstract form only, also show sustained benefits in *off* time reduction and tremor‐, rigidity‐, and bradykinesia‐subscores in the medication *off* condition, the latter being more prominent after STN‐DBS compared with GPi‐DBS.^24^

**FIG 1:**
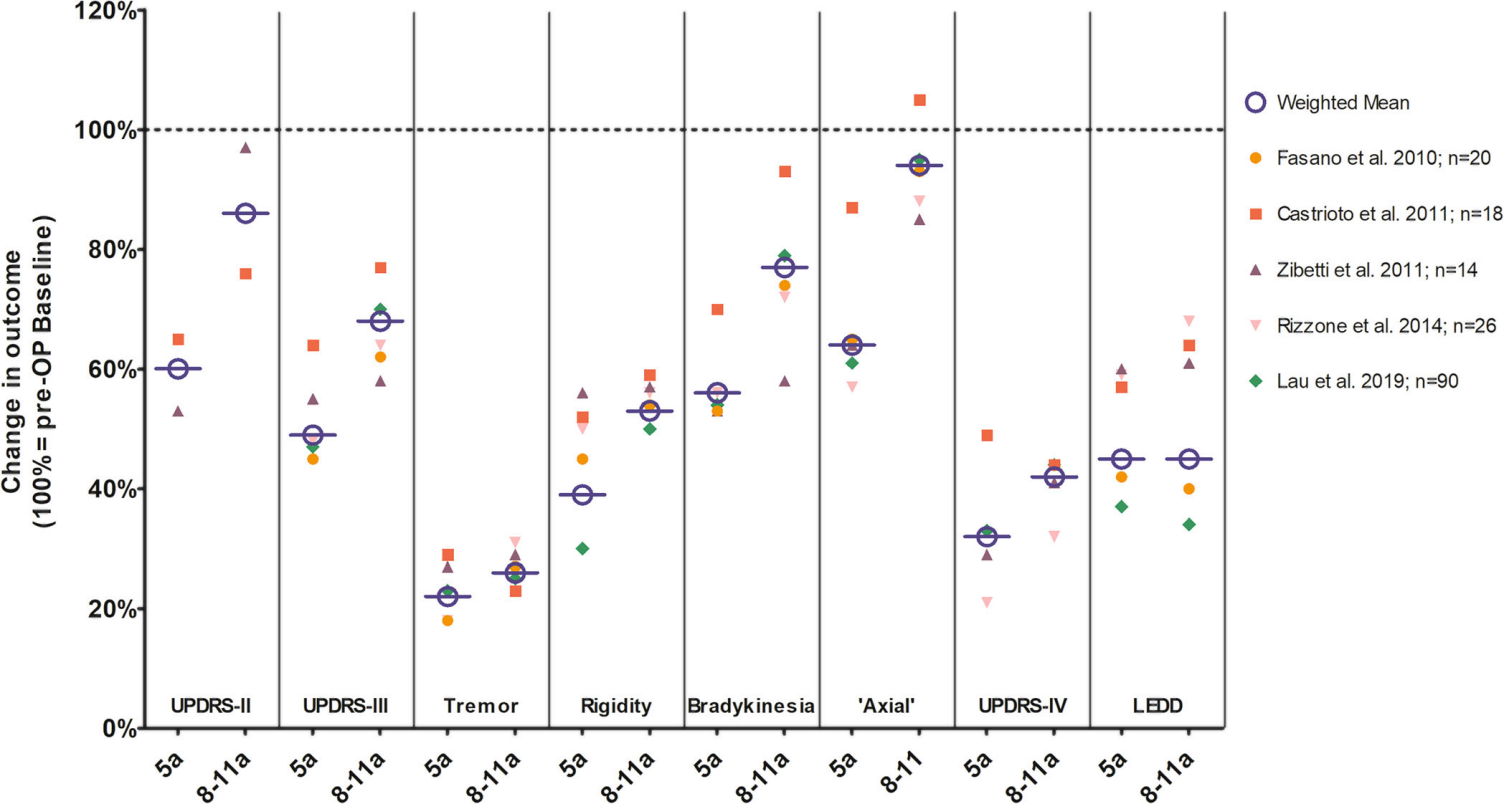
Responsiveness of PD symptoms to long‐term treatment with STN‐DBS. Studies reporting outcomes at two time points (ie, 5 years of follow‐up and beyond [range, 8–11 years]) are included for illustrative reasons and for better comparability of changes over long periods of follow‐up. Results are shown as percentage change from preoperative baseline indicated by the 100% line to no symptoms at 0%. The results of individual studies are represented by dots and the circles with bars show weighted mean values across studies (total n = 168). Activities of daily living scores (UPDRS‐II) and motor scores (UPDRS‐III) as well as motor subscores for tremor, rigidity, bradykinesia, and combined “axial” motor symptoms are measured in the stimulation ON and in the practically defined medication *off* state after overnight withdrawal (ie, >12 h) of dopaminergic medications. In addition, changes in motor fluctuations and dyskinesias (UPDRS‐IV) and in LEDDs are shown. LEDD = levodopa‐equivalent daily doses; PD = Parkinson's disease; UPDRS = Unified Parkinson's Disease Rating Scale. [Color figure can be viewed at wileyonlinelibrary.com]

These findings are consistent with those from multiple observational long‐term studies reporting sustained effects on motor outcomes of STN‐DBS over periods of 8–16 years.^25^, ^26^, ^27^, ^28^, ^29^, ^30^, ^31^, ^32^, ^33^ Figure 1 shows changes of outcome measures of those studies that reported follow‐up results at 5 years and at 8–11 years in comparison to preoperative baseline motor functioning. These suggest that STN‐DBS induced improvements of cardinal motor symptoms (tremor>rigidity>bradykinesia)^29^ are sustained in the long‐term with reductions from baseline in *off* medication scores of 30%–50%. Effects on levodopa‐induced motor complications also persist in the long‐term with improvements of 60%–70% and are accompanied by dose reductions of dopaminergic medications in the order of 40%–60% compared with the preoperative state.^25^, ^26^, ^28^, ^29^, ^30^, ^32^, ^33^ Nevertheless, improvements of motor scores become blunted with increasing duration of follow‐up (Fig. 1) and *on* medication motor scores generally decline below baseline levels by year 5.^29^, ^34^ Similarly, DBS effects on ADL persist at least to year 5 for *off*‐medication assessments, whereas *on*‐scores are usually worse than baseline by that time.^25^, ^28^, ^33^, ^34^ Dysarthria, freezing, and impaired gait are generally less responsive to stimulation or may even sometimes be worsened by stimulation itself.^34^ Across the different long‐term series, these functions deteriorate to the pre‐operative state or below within 9 years of follow‐up.^25^, ^27^, ^29^, ^32^, ^33^

Although long‐term observations in GPi‐DBS‐treated patients are limited, available 5‐ to 8‐year data suggest that beneficial effects in terms of motor fluctuations and dyskinesia may be similar to those reported for the STN target.^35^, ^36^

Overall, these observations strongly suggest that DBS can improve cardinal motor features and control levodopa‐related motor complications for 10 years and longer, which is remarkable for patients that, on average, already had disease durations of more than 10 years at the time of surgery. In analogy to the “levodopa honeymoon” period, where PD patients enjoy the full benefit from levodopa before the occurrence of motor complications, it has been argued that DBS leads into a “second treatment honeymoon.”^37^ Nonetheless, overall *on* motor function, particularly axial features, and ADL‐scores deteriorate below pre‐surgical levels within 5 years following DBS surgery, consistent with continued disease progression.

Non‐motor complications associated with chronic dopaminergic therapy may also be reduced following STN‐DBS, with one long‐term follow‐up study showing lasting reduction of impulse control disorders and mood fluctuations.^38^ On the other hand, chronic STN‐DBS can be associated with apathy,^38^ possibly related to the marked reductions of dopaminergic medication following DBS. Such changes related to dopaminergic medication may be much less evident in GPi‐DBS.

In the few studies providing information on the long‐term outcome of QoL measures, initial improvement for the first 3 years of treatment is followed by a decline to baseline levels 5 years into DBS treatment.^39^, ^40^ Although the long‐term data on QoL after DBS may be too limited for firm conclusions, they seem to suggest that the QoL effects of sustained control of levodopa‐related motor complications may become superseded by other facets of motor and non‐motor decline over time.

## Effects of DBS on the Progression of Disability in PD

The major unmet need in the management of PD is to slow disease progression and reduce or prevent key disability milestones that characterize late stage disease and are resistant to current treatments.^41^, ^42^ Long‐term follow‐ups of the Sydney multicenter cohort suggest that, after 15–20 years of disease duration, >80% of patients will have developed recurrent falls, >50% will suffer from hallucinations and/or dementia, and >40% will have been placed in a nursing home.^5^, ^6^ By such time levodopa induced motor complications affect almost all patients, but are usually not considered a leading cause of disability anymore.^7^ In the very advanced stages of the disease a set of disability milestones including psychosis, falls, dementia, and institutionalization tend to cluster together, preceding death by ~3–5 years — a process that seems to be independent of age at disease onset, disease duration, levodopa‐response, and age at death.^7^ A comprehensive meta‐analysis of 18 studies found increased mortality in PD patients versus controls, with a pooled mortality ratio of 1.5 and survival rates reduced by 5% per year.^43^ Although dopaminergic therapies can effectively control motor symptoms, no agent has yet been shown to modify underlying disease progression or normalize life expectancy.^3^, ^42^, ^43^

For DBS, experimental studies in different animal models seem to suggest “neuroprotective” effects.^20^, ^21^ Earlier studies, for instance, found an increased survival of dopaminergic neurons after several weeks of STN‐DBS treatment in toxin‐mediated PD models in nonhuman primates^44^ or in rodents.^45^ Such effects were not evident in a study using stimulation of the rodent correlate of the GPi (the entopeduncular nucleus).^46^ Toxin‐based models may not adequately reflect the molecular pathology of human PD and some subsequent studies used α‐synuclein‐based animal models. In a study in human wild‐type α‐synuclein‐overexpressing PD rat model STN‐DBS did not protect against forelimb akinesia, striatal denervation, or nigral neuronal loss.^47^ A more recent study in a human A53T mutated α‐synuclein‐overexpressing PD rat model, however, showed a sparing of dopaminergic nigral neurons in rats that were treated with STN‐DBS over 3 weeks versus a control group that was implanted, but kept OFF stimulation.^48^ Moreover, this was paralleled by improvements in motor deficits that were maintained after 24 hours spend in the OFF stimulation condition. Potential mechanism for such observations in various animal models include reversal of pathologic basal ganglia oscillations, reducing excitotoxicity arising from overactive glutamatergic projections from the STN, and/or by increasing levels of brain‐derived neurotrophic factor.^20^, ^21^, ^49^

These studies have prompted speculations that, on top of providing sustained symptomatic effects, STN‐DBS might also modify the long‐term progression of PD.

### Does DBS Modify the Underlying Progression of PD?

In the context of this review — as in regulatory science — the term “disease‐modification” is used to mean that a therapy like DBS is capable of positively influencing the course of the disease beyond its symptomatic effects. Although “neuroprotection” itself cannot clinically be demonstrated in humans, the assessment of PD motor scores in the “practically defined” *off* condition has gained acceptance as a surrogate marker of the underlying severity of the disease, enabling comparisons of long‐term decline of motor function and therefore explore the existence of “disease‐modification”.

**FIG 2:**
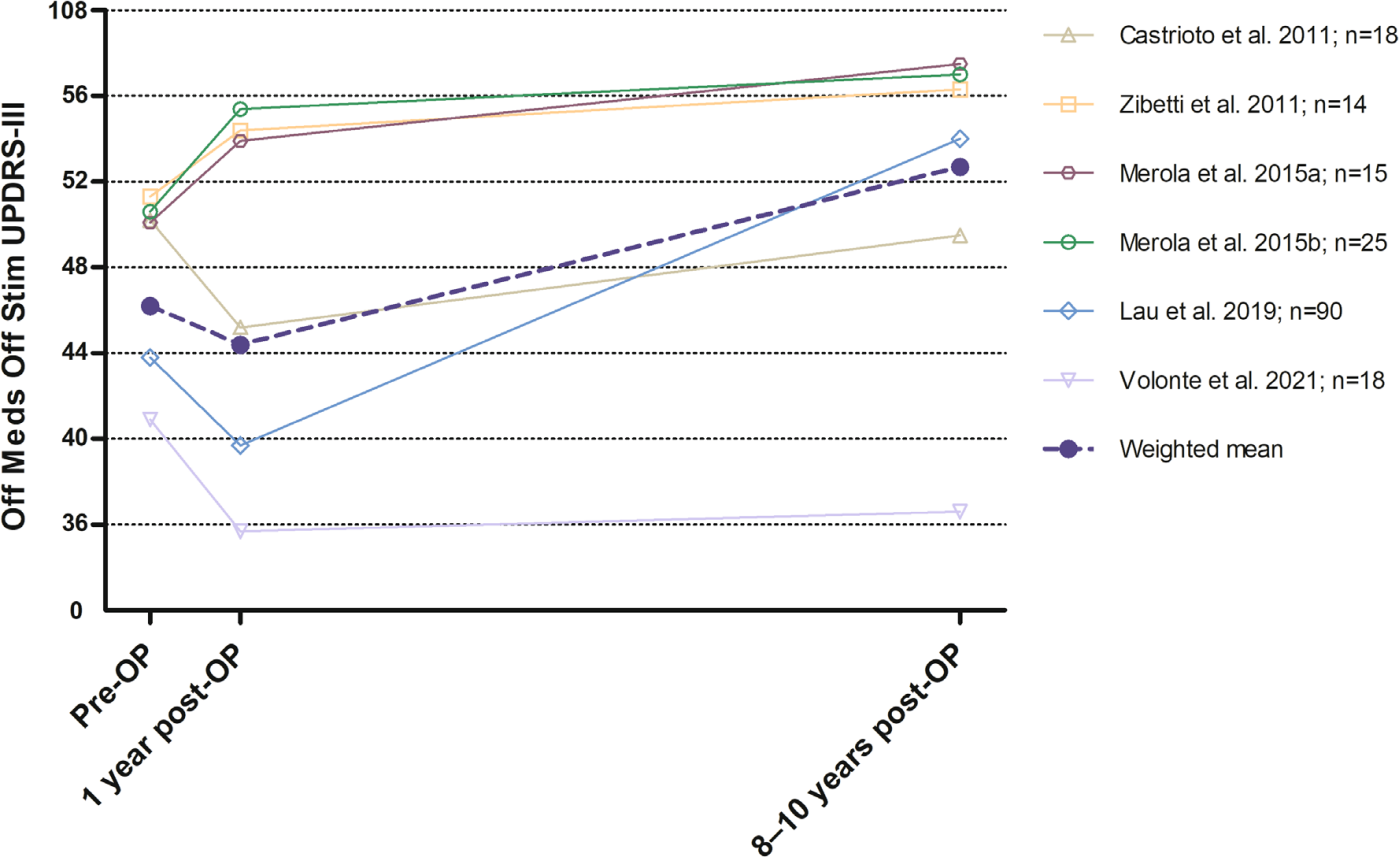
Progression of *off* medication, OFF stimulation motor scores in long‐term observational studies >5 years. All patients included in these long‐term studies (total n = 180) had subthalamic stimulation and were assessed in the practically defined *off* state after overnight withdrawal (ie, >12 h) of dopaminergic medications and at least 40 min after switching stimulation OFF. The weighted mean UPDRS‐III motor scores were 46.2 points pre‐surgery, 44.4 points 1 year post‐surgery, and 52.7 points at last follow‐up (8–10 years post‐surgery). Therefore, excluding the drop in UPDRS‐III scores seen up to year 1 (−1.8 points), there was an UPDRS‐III increase of 8.3 points from post‐surgery to last follow‐up, equaling a ~1.0 points increase per year. UPDRS‐III = Unified Parkinson's Disease Rating Scale motor score. [Color figure can be viewed at wileyonlinelibrary.com]

Several longitudinal cohort studies in STN‐DBS patients have reported serial assessments of *off* medication and OFF stimulation motor scores post‐DBS after 1 year and after 8–10 years of follow‐up as compared to pre‐DBS *off* medication scores (Fig. 2 and Supplementary Table S1).^25^, ^29^, ^30^, ^33^, ^50^ Variability across studies is considerable, but all show deterioration from year 1 after DBS until last follow‐up. Calculating the average rate of change, we found an annual worsening of 1.0 point per year for this time period. This is lower than *off* medication UPDRS‐III increments reported by available studies in conventionally managed advanced patients, which are between ~1.4–2.6 points per year.^51^, ^52^ However, patients in these medically treated cohorts were followed somewhat earlier in their disease course compared to the DBS‐treated patients analyzed in Figure 2, which is of relevance as motor decline and indeed nigral cell loss may follow an exponential curve with faster progression earlier in comparison to later in the disease.^1^, ^53^

The largest, most detailed, and rigorous of the studies included in Figure 2 also assessed progression in single motor domains over 10 years and found that in the *off* medication and OFF stimulation condition deterioration was fastest for axial motor symptoms, which were also the strongest predictor of death, followed by bradykinesia and rigidity, whereas tremor was still improved.^29^ The latter observation is in line with a smaller blinded study in 18 patients.^33^

Another study included in Figure 2 explored the impact of DBS on disease progression by retrospectively constructing a “delayed‐start” paradigm through comparing outcomes between patients with a Hoehn and Yahr *on*‐medication stage of <3 and duration of motor fluctuations of ≤3 years at the time of surgery versus patients operated on later in the course of their disease.^50^ Eight years after DBS implantation both patient groups (15 “Early‐Stim” versus 25 “Late‐Stim”) had similar declines in UPDRS‐III scores, but “early” patients still had better ADL function as determined by UPDRS‐II scores compared with their pre‐surgery baseline, whereas UPDRS‐II scores of “late” patients had deteriorated below baseline.^50^

Unfortunately, all of the above studies are limited by their observational and uncontrolled designs and high drop‐out rates owing to the long follow‐up. Potential carry‐over of stimulation effects and the levodopa long‐duration response^51^ additionally limit conclusions from stimulation OFF assessments. Indeed, numerous reports on the DBS withdrawal syndrome following accidental cessation of chronic stimulation (eg, because of battery depletion) document the rapid recurrence of severe akinetic‐rigid symptoms^54^ arguing against clinically relevant modifying effects of chronic DBS on underlying disease progression. In line with this, the few studies that have assessed biomarkers of disease progression in DBS treated PD patients also failed to detect signals of potential disease‐modification: an uncontrolled 18F‐fluorodopa positron emission tomography study reported annual rates of decline of striatal dopaminergic tracer uptake of 10%–12% in 30 STN‐DBS treated PD patients in the first 1–2 years after implantation that was within the range of previously reported longitudinal imaging studies in medically managed patients.^55^ In addition, a recent post mortem study provided no evidence for improved neuronal survival, reduced nigral pathology, or increased striatal dopamine and dopamine metabolites in 11 STN‐DBS‐treated PD patients versus 22 matched PD patients on conventional therapies.^56^, ^57^

In summary, there is currently no evidence from clinical studies that DBS would exert modifying effects on the underlying neurobiological progression of PD. A broad and pragmatic definition of “disease‐modifying” might however, also include the effects of symptomatic therapies as far as they reduce the severity and functional impact of motor and non‐motor symptoms and therefore, exert beneficial effects on the progression of clinical disability.

### DBS Effects on Progression to Disability Milestones

Randomized controlled and prospective studies to test possible effects of DBS on the evolution of key disability milestones and on overall survival are not available and will hardly be feasible given the observational periods involved. Nevertheless, there are multiple uncontrolled long‐term studies reporting frequencies of key disability milestones in STN‐DBS patients (Supplementary Table S2).^25^, ^26^, ^27^, ^29^, ^31^, ^32^, ^58^, ^59^, ^60^, ^61^ At follow‐up durations of 8–12 years after DBS implantation, average rates of motor disability milestones were 52% for dysarthria, 26% for dysphagia, 77% for freezing of gait, and 61% for falls. Disabling non‐motor symptoms included psychosis in 48% of patients, depression in 48%, dementia in 38%, and apathy in 46% and 32% of patients were institutionalized. In one large observational study specifically addressing dementia, incidence rates seen were similar to those reported in the general PD population.^62^ Given the long mean overall disease durations of >20 years in these DBS cohorts, these numbers seem to compare favorably with those reported from the only available long‐term follow‐up study of medically managed PD patients with only slightly shorter disease duration.^5^, ^6^ However, DBS candidates generally represent a PD subpopulation of younger age with fewer comorbidities (and without on‐period freezing or dementia that are regarded as exclusion criteria for DBS). Therefore, it is impossible to conclude on potential DBS effects on delaying disability milestones without data from matched PD controls.

There are only a few controlled studies that have tried to design retrospectively matched control groups without DBS as a comparator and most have assessed survival only (see next section). One of these studies, however, also assessed nursing home placement and found a markedly reduced risk in STN‐DBS treated patients with an odds ratio of 0.1.^63^ Another controlled retrospective long‐term study found a significantly lower risk for recurrent falls (hazard ratio [HR], 0.57) and for psychosis (HR, 0.26)^64^ and a third shorter‐term study over 3 years also found a similarly reduced risk for falls with STN‐DBS.^65^ Postoperative progression on Hoehn and Yahr scores does not, however, seem to be different in STN‐DBS treated versus medically managed patients^64^ and the beneficial impacts seen on institutionalization, falls, and psychosis may well be mediated through symptomatic DBS effects with improved motor symptom control and reduction in dopaminergic therapies, rather than true “disease modification”.

### DBS Effects on Survival

**FIG 3:**
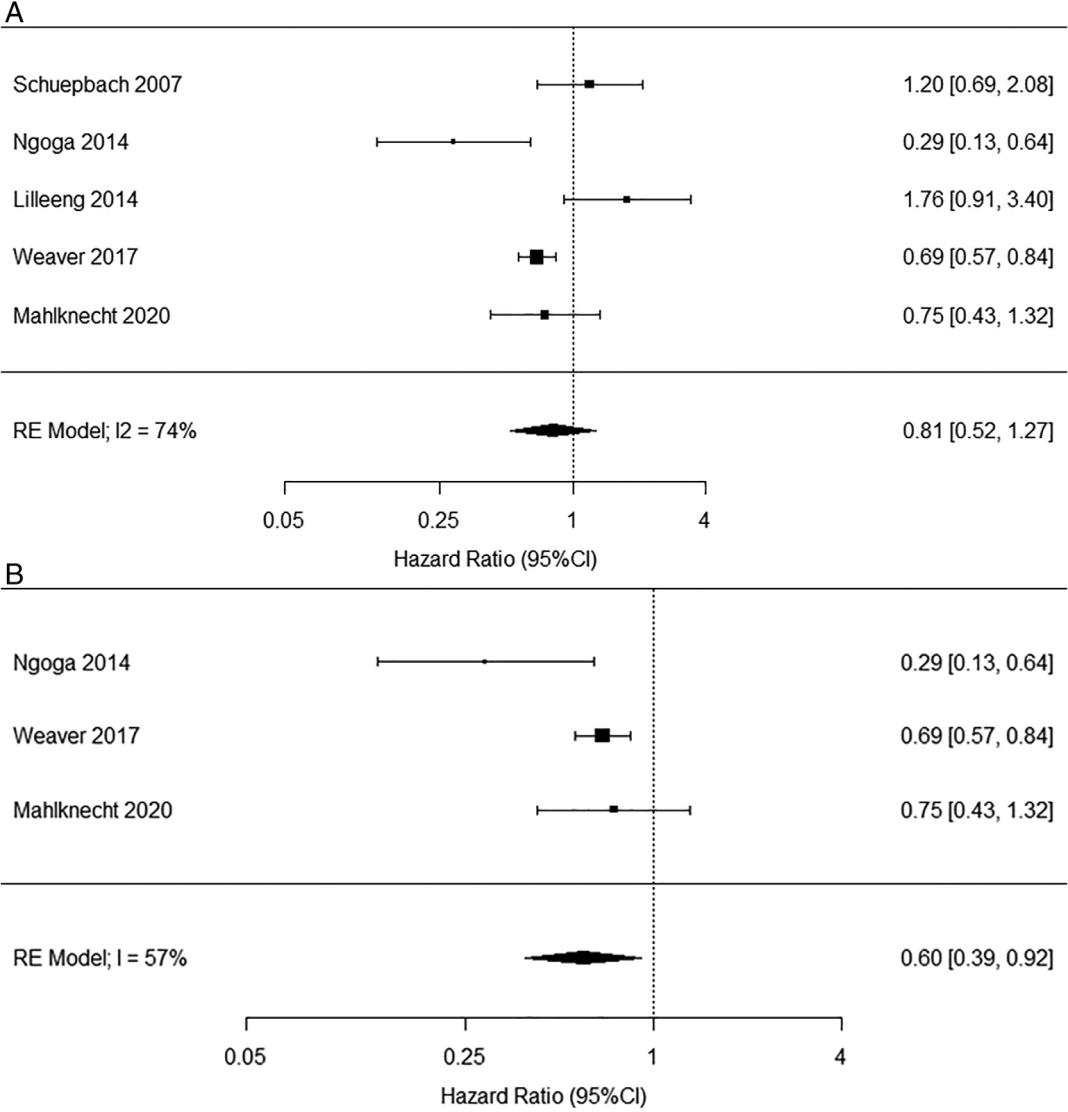
Meta‐analysis of controlled studies on survival in PD patient with versus without DBS. Across all five studies (upper panel A), STN‐DBS was associated with a trend for increased survival that was not statistically significant with substantial heterogeneity as per I2 index. Two of these studies have used controls from historical cohorts and there is no reporting of balancing patient groups or statistical adjustments according to important confounders such as comorbidities, age of onset, disease duration, or severity.^66^, ^67^ Excluding these from the meta‐analysis (lower panel B) results in a significant survival benefit with DBS with a lower, but still substantial heterogeneity. Meta‐analysis was calculated with R software (version 3.6.3; R Foundation for Statistical Computing, Vienna, Austria) using the metaphor package (Random Effect Model).

A total of five studies have looked at survival after DBS in a controlled fashion (Supplementary Table S3)^63^, ^64^, ^66^, ^67^, ^68^ and a meta‐analysis of these is presented in Figure 3. Overall, DBS was associated with a trend for increased survival (although not statistically significant). Excluding the two studies, which used controls from historical cohorts without matching or statistical adjustments for important confounders,^66^, ^67^ results in a HR of 0.60 (95% CI, 0.39–0.92). Although the remaining studies have made substantial efforts to construct adequate control PD populations,^63^, ^64^, ^68^ they still suffer from limitations that are inherent to their retrospective design including insufficient adjustments for baseline confounders such as comorbidities or motor severity in one study^63^ or disease duration/severity in another.^68^ Nevertheless, the data seem to point to a potential survival benefit in favor of DBS. The largest of the studies calculated a mean gain in lifetime of 7.6 months.^68^ This small survival benefit may reflect improved motor control in DBS patients, which may in turn positively influence general health (eg, increased mobility, better swallowing and respiratory functions, and more efficient personal care). Moreover, DBS patients are likely to have more frequent appointments and contact with medical teams including physiotherapy, occupational therapy and speech therapy, which might further contribute to such effects.

On the negative side, suicides can be a rare (<1%), but alarming side effect of stimulation. A recent long‐term observational study in a large sample of STN‐DBS treated patients found an elevated rate of suicides and suicidal behavior over the first 3 postoperative years, but not thereafter.^69^

## Is Earlier Better?

Although there is no solid evidence to support DBS having effects on the underlying progression of PD pathology, available studies convincingly show that for those with advanced disease and disabling motor complication the initiation of DBS translates into a gain of at least 5 years of recovered motor control and associated improvements in QoL. Available data are almost exclusively from patients that were operated after more than 10 years of disease, that is, at a time when disease progression has already led to significant impairments affecting many aspects of daily living including mobility, social adjustment, and professional activity, with corresponding loss of QoL. Therefore, it has been argued that initiating DBS earlier, as soon as motor fluctuations appear, may optimize both short and long‐term outcome.^70^ Earlier DBS also entails operating on younger and fitter patients with lower surgical risks.

The EARLYSTIM trial^14^ supports short‐term gains over medical management regarding QoL, motor function (including freezing of gait),^71^ ADLs, and behavioral complications of dopaminergic medication.^72^ Although the trial has been criticized because of its unblinded nature with potential placebo and lessebo‐related effects,^73^ it has had an impact on clinical practice further supporting a trend toward progressively earlier surgical selection.^50^ Longer‐term outcomes of the EARLYSTIM cohort will be critical to clarify the long‐term advantages of performing STN‐DBS in PD patients with early motor complications and its impact on further disease progression. Interestingly, a 10‐year follow‐up study of the EARLYSTIM pilot trial^74^ published in abstract form found that all of the patients initially randomized to BMT had eventually undergone DBS at varying delays.^75^ At last follow‐up, there were no differences between early and delayed DBS groups regarding motor function, ADLs, QoL, mood, or cognitive function.

In the Vanderbilt trial, researchers went even further and randomized 30 early PD patients without motor complications to 24 months of STN‐DBS and BMT or BMT alone. The study failed to detect differences between treatment arms for both the primary (motor worsening after 1 week of stimulation and medication washout or change in levodopa equivalent dose from baseline) and multiple motor and QoL‐related secondary outcomes,^76^ but two of the 15 operated patients had serious surgery‐related adverse events. This highlights the fact that using DBS in early PD patients involves exposure to significant surgical risks, which cannot be easily justified against a background of a relatively low level of pre‐surgical PD disability. In addition, diagnostic error in distinguishing PD from other forms of degenerative parkinsonism is not uncommon in early disease stages and may introduce another scenario of unnecessary risk and cost related to DBS.^77^ Nevertheless, in a 5‐year follow‐up, the group reported advantages in stimulated patients regarding need for and complexity of PD medications and severity of rest tremor and announced the conduction of a multicenter, phase‐III trial evaluating DBS in early PD (IDEG050016).^78^

**FIG 4:**
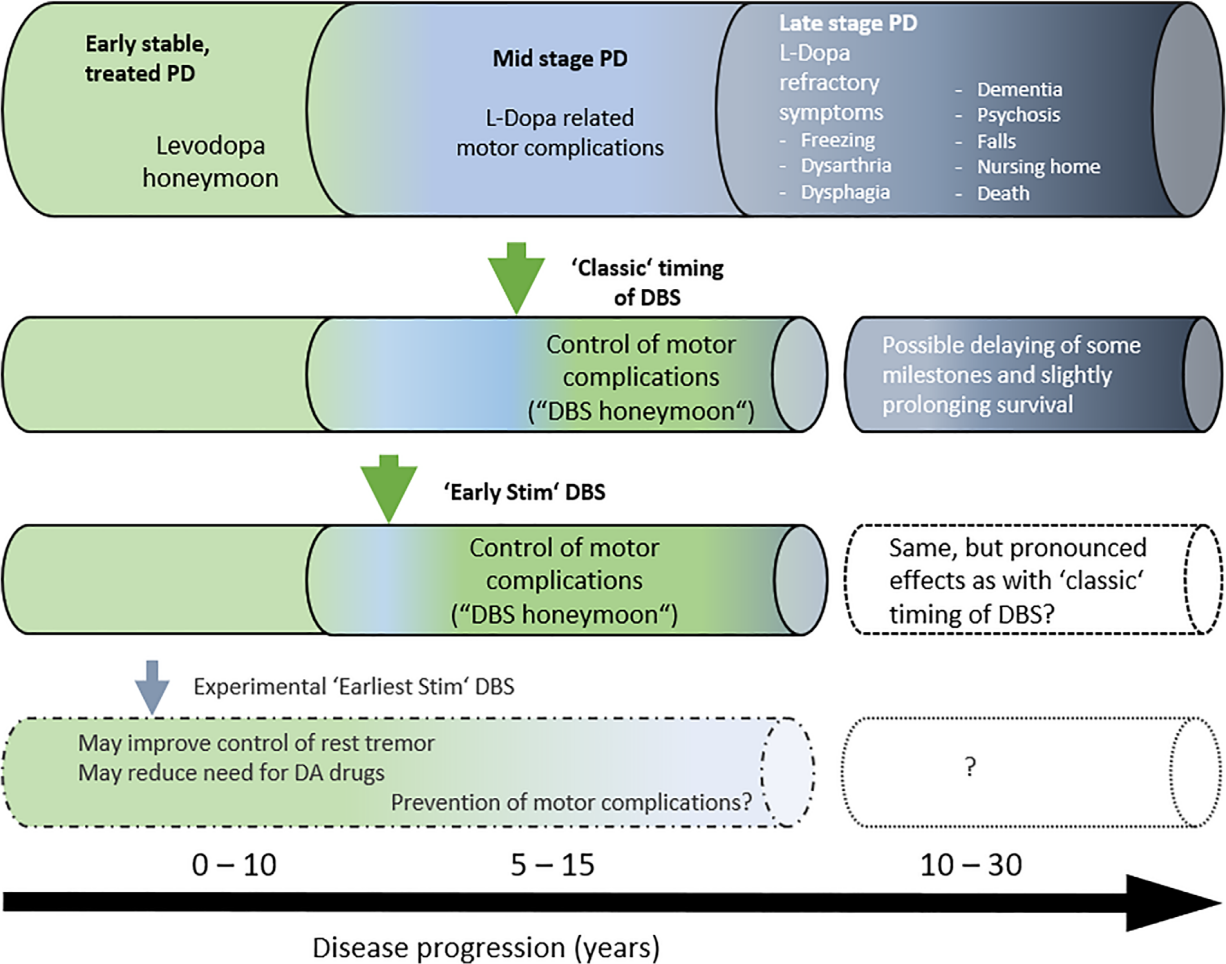
Effects of STN‐DBS on motor symptoms and potential effects on late‐stage disability milestones and disease progression. Effects of STN‐DBS are shown in relation to the natural history of PD under conservative treatments without DBS (upper row) and according to the timing of DBS introduction. The second row “classic timing of DBS” refers to the majority of PD patients with DBS, who are operated when motor fluctuations and dyskinesias have led to substantial disability. In past clinical trials, and also observational studies (see Table 1 and Supplementary Tables S1 and S2) such patients are approximately 60 years of age, have mean disease durations of 10–13 years, and a long‐standing history of motor complications. The third row refers to the EARLYSTIM trial, where younger patients with a mean age of 52 years (<60 years), shorter mean disease duration of 7.5 years and motor complication history (<3 years) were included (Table 1). This trial has led to a trend toward progressively earlier surgical selection also in clinical practice. However, no follow‐up of the EARLYSTIM cohort has yet been published and the effects of earlier surgery on the very long‐term outcomes remain unclear. The last row “earliest stim DBS” refers to experimental use of DBS in early PD patients that do not yet experience motor complications. We refer to the main text for more details. [Color figure can be viewed at wileyonlinelibrary.com]

Figure 4 illustrates established and putative effects of STN‐DBS on the course of PD as a function of the timing of DBS introduction.

## Conclusions and Future Directions

The introduction of STN‐DBS by Benabid and Pollak in the early 1990s clearly marks the second major breakthrough in the symptomatic treatment of PD after the disovery of levodopa in the 1960s. Since then, many RCTs and a growing body of observational evidence have established the profound and long‐lasting symptomatic effects of DBS for 10 years and longer. Therefore, DBS has clearly has made an impact on the course of PD, not only by substantially diminishing levodopa induced motor complications, but also by providing relief for those with drug‐refractory tremor. Additionally, although in a less predictable way, STN‐DBS can also help to control dopaminergic neuropsychiatric side effects such as impulse control disorders, the presence of which has recently been proposed as an indication for DBS per se.^79^ These effects and the resultant improvements in QoL and ADLs, however, start to decline around 5 years into DBS treatment as disease progression begins to catch up. Although motor symptoms including tremor and bradykinesia are well‐controlled by DBS for 10 years or longer, axial motor symptoms like gait impairment, freezing of gait, and dysarthria worsen and, along with bothersome non‐motor symptoms such as psychosis, dementia, and dysautonomia dominate the clinical picture seen in patients with very long‐term DBS.^80^

Whether DBS is able to delay such major disease milestones or modify the progression of the disease is difficult to answer from currently published observational studies because of their heterogenous and mainly non‐controlled designs. Nonetheless, there are signals from studies comparing DBS treated patients with retrospectively constructed control PD populations suggesting that chronic subthalamic DBS may lower the risk for or delay some important disability milestones such as falls, psychosis, and need for long‐term care and may be associated with slightly prolonged survival. Reasons behind this may relate to the long‐term control of motor complications and reductions in medication‐induced side effects, which lead to improved mobility, personal care, and general health, rather than to a true “disease‐modifying” effect. A definite answer to these fundamental questions would require RCTs of early versus later DBS separated by sufficient delays or of DBS versus BMT of sufficiently long follow‐up periods. Such trials would be extremely challenging to implement and in reality appear hardly feasible, such that long‐term registry studies the best alternative.

Further progress may be achieved with patient selection for DBS based on better understanding and identification of disease subtypes with differential response to neurostimulation.^81^ Recently, different PD phenotypes have been described: the “malignant PD” type characterized by higher motor deficits and non‐motor symptom burden in terms of cognitive impairment, rapid eye movement (REM) sleep behavior disorder, and dysautonomia; the benign “mild motor‐predominant PD” type; and an intermediate type.^82^, ^83^ The malignant PD phenotype is associated with faster progression and higher risk for major disease milestones and death. In studies assessing different DBS effects in these subtypes, the malignant PD type has been associated with faster loss of independence in daily life irrespective of PD onset, PD duration, and motor improvement with stimulation.^84^ Additionally, studies in genetic forms of PD have reported correlations with DBS outcomes. Specifically, some *LRRK2* mutation variants seem to predict good DBS outcomes, similar to sporadic PD patients.^85^ In contrast, glucocerobrosidase (*GBA*) gene variants have been associated to poorer DBS outcomes in terms of cognitive decline and non‐motor symptom burden, but not response of motor symptoms to DBS.^85^, ^86^ According to a recent multicenter observational study, cognition in STN‐DBS treated PD patients with *GBA* mutations deteriorates faster compared with *GBA* patients without DBS, suggesting that patients should be screened for *GBA* variants as part of the pre‐surgical work‐up and counseled accordingly.^87^ Beyond this latter study, however, there are no studies assessing patients with specific PD subtypes or genetic mutations treated with DBS and comparing their outcome to the patient groups with the same phenotype or genotype that are not treated by DBS. Hence, DBS effects within certain patient groups are still unknown.

Meanwhile, technological developments are rapidly advancing and enabling new neurostimulation approaches. Examples include directional electrodes, artificial intelligence and imaging‐based programming, and adaptive and closed‐loop stimulation, that are aimed at further refining stimulation toward personalized treatment and will assist clinicians to deal with increasingly complex programming features.^88^ For patients already under DBS, strategies to improve outcomes are also being investigated. One example are axial motor symptoms such as gait impairment and freezing of gait for which low frequency stimulation,^89^, ^90^ drugs (eg, rivastigmine),^91^ or physiotherapy may provide relief.

Therefore, DBS will continue to evolve, leading toward enhanced efficacy and safety. The latter issues are what matter most to patients and even if DBS does not ultimately prevent progressive disability, it continues to substantially change the outlook for many people with PD, whose function and QoL has become compromised by motor complications.

## Financial Disclosures

P.M. has received a grant from the “Tiroler Wissenschaftsfond” and a speaker's honorarium from Boston scientific.

T.F. has received grants from National Institute of Health Research, Edmond J. Safra Foundation, the Michael J. Fox Foundation, John Black Charitable Foundation, Cure Parkinson's Trust, Innovate United Kingdom (UK), Janet Owens Research Fellowship, Rosetrees Trust, Van Andel Research Institute, and Defeat MSA. He has served on advisory boards for Peptron, Voyager Therapeutics, Handl therapeutics, Living Cell Technologies, Bial, and Profile Pharma. He has received honoraria for talks sponsored by Bial, Profile Pharma, and Boston Scientific.

P.L. has received grants from National Institute of Health Research and Boston Scientific. She has received honoraria for talks or courses sponsored by Medtronic and Boston Scientific.

W.P. reports personal fees from AbbVie, Affiris, AstraZeneca, BIAL, Boston Scientific, Britannia, Intec, Ipsen, Lundbeck, Neuroderm, Neurocrine, Denali Pharmaceuticals, Novartis, Orion Pharma, Teva, UCB, and Zambon. He reports consultancy and lecture fees in relation to clinical drug development programs for Parkinson's disease and has received grant support from The Michael J. Fox Foundation and the EU FP7 and Horizon 2020 programs.

## Conflict of Interest

The authors report no conflict of interest related to this work.

## Author Roles

(1) Research project: A. Conception, B. Organization, C. Execution; (2) Statistical Analysis: A. Design, B. Execution, C. Review and Critique; (3) Manuscript: A. Writing of the First Draft, B. Review and Critique.

P.M.: 1A, 1B, 1C, 2A, 2B, 2C, 3A, 3B

T.F.: 1A, 1C, 2C, 3B

P.L.: 1A, 1C, 2C, 3B

W.P.: 1A, 1B, 1C, 2C, 3A, 3B

## Supporting information


**Appendix S1** Supporting information

## Data Availability

No original data were used. The data extracted from the literature review is illustrated in Table 1, Figures 1 to 3, and the 3 supplementary tables.
