# Supplementary material for: How Does Deep Brain Stimulation Change the Course of Parkinson's Disease?
Source: Mov Disord. 2022 May 12;37(8):1581–92. doi: 10.1002/mds.29052 (PMC9545904; doi:10.1002/mds.29052)
Supplement: Supplementary file 1 — Appendix S1 Supporting information [file MDS-37-1581-s001.docx]

**Appendix – Supplementary Tables**

**Supplementary Table 1: Long-term (>5 years) longitudinal studies assessing motor functioning off medication and off stimulation**

| **Study** | **N** | **Mean age at baseline** | **Mean disease duration at baseline** | **FU**  **(y)** | **Minimum**  **washout period** | **Results (UPDRS-III off med, off stim)** | | | **Significance of changes over time / remarks** |
| --- | --- | --- | --- | --- | --- | --- | --- | --- | --- |
|  |  |  |  |  |  | **Pre-OP** | **1 y post-OP** | **Last FU** |  |
| Castrioto et al. 2011^1^ | 18 | 53 ± 8 | 13 ± 5 | 10 | Meds overnight withdrawal  60 min for stim | 50.2 ± 13.3 | 45.2 ± 11.8 | 49.5 ± 10.3 | Not significant |
| Zibetti et al. 2011^2^ | 14 | 60 ± 7 | 17 ± 5 | 10 | Meds overnight withdrawal  60 min for stim | 51.3 ± 15.4 | 54.4 ± 10.9 | 56.3 ± 18.4 | Not significant |
| Merola et al. 2015^3^ | 15 ‘Early-Stim’ | 56 ± 7 | 12 ± 3 | ≥8 | 12 h for meds  40 min for stim | 50.1 ± 14.5 | 53.9 ± 13.0 | 57.5 ± 12.7 | Significance of within group change not given; no significant difference between groups |
|  | 25 ‘Late-Stim’ | 61 ± 4 | 17 ± 5 |  |  | 50.6 ± 15.0 | 55.4 ± 10.9 | 57.0 ± 11.1 |  |
| Lau et al. 2019^4^ | 90 | 56 ± 9 | 13 ± 6 | 10 | 12 h for meds  90 min for stim | 43.8 ± 15.8 | 39.7 ± 15.6 | 54.0 ± 14.7 | Significant worsening; subscores for akinesia, rigidity and axial symptoms increased, while tremor decreased; axial disability most strongly indicated increased risk of death |
| Volonte et al. 2021^5^ | 18 | 56 ± 7 | 11 ± 4 | 9  (11; n=11) | 12 h for meds  n.g. for stim | 40.9 ± 11.2 | 35.7 ± 9.6 | 36.6 ± 10.1  (45.3 ± 17.6) | Not significant |

In all studies subthalamic deep brain stimulation was used.

Abbreviations: UPDRS-III = Unified Parkinson’s disease rating scale motor section

**Supplementary Table 2: Descriptive results of long-term (>5 years) observational studies reporting disease milestones in DBS treated PD patients**

| **Study** | **Year of DBS** | **N** | **Cohort characteristics (means/medians)** | | | **Major motor disease milestones** | | | | | **Major non-motor disease milestones** | | | | **Nursing home** | **n died /**  **n total** |
| --- | --- | --- | --- | --- | --- | --- | --- | --- | --- | --- | --- | --- | --- | --- | --- | --- |
|  |  |  | **Age at DBS** | **Disease duration at DBS** | **FU duration** | **Dysarthria** | **Dysphagia** | **FOG** | **Falls** | **Psychosis** | | **Depression** | **Dementia** | **Apathy** |  |  |
| Fasano et al. 2010 ^6^ | 1996 – 2001 | 20 | 57 ± 7 | 14 ± 5 | 8 | 8  (40%) | 1  (5%) |  |  | 4  (20%) | | 5  (25%) | 1  (5%) | 4  (20%) |  | 3 / 28  (11%) |
| Zibetti et al. 2011 ^2^ | 1998–2002 | 14 | 60 ± 7 | 17 ± 5 | 10 |  |  |  |  |  | |  | 4  (29%) |  |  | 10 / 24  (42%) |
| Castrioto et al. 2011 ^1^ | 1996–2000 | 18 | 53 ± 8 | 13 ± 5 | 10 | 11  (61%) | 2  (11%) |  |  | 5  (28%) | | 3  (17%) | 4  (22%) |  |  | 12 / 41  (29%) |
| Merola et al. 2011 ^7^ | 1998–2006 | 14 | 61 ± 6 | 23 ± 2  (>20 years) | >7 | 9  (64%) | 12  (86%) | 9  (64%) | 9  (64%) | 9  (64%) | | 6  (43%) | 6  (43%) |  |  | NA |
| Rizzone et al. 2014 ^8^ | 1998–2001 | 26 | 58 ± 7 | 15 ± 6 | 11 | 14  (54%) | 3  (12%) | 18  (69%) | 17  (65%) | 5  (19%) | | 6  (23%) | 6  (23%) | 5  (19%) |  | 14 / 69  (20%) |
| Henriksen et al. 2016 ^9^ | 1998–2003 | 79 | 60 ± 8 | 16 ± 6 | 9 | 27  (34%) | 21  (27%) |  |  | 46  (58%) | | 47  (60%) | 36  (46%)* |  | 32  (42%) | 24 / 79  (30%) |
| Constantinescu et al. 2017 ^10^ | 1993 – 2001 | 23 | 60  (48-71)* | 18  (10-28)* | 12 | 12  (52%) | 8  (35%) |  |  | 14  (61%) | | 16  (70%) | 14  (61%) |  | 12  (52%) | 14 / 23  (60%) |
| Lilleeng et al. 2015 ^11,12^ ** | 2001–2006 | 16 | 61 ± 8 | 13 ± 5 | 6–9 |  |  | 9  (56%) | 8 (50%) | 8  (50%) | | 1  (6%) | 5  (31%) | 7  (44%) |  | 12 / 28  (43%) |
| Lau et al. 2019^4^ | 1996–2003 | 90 | 56 ± 9 | 13 ± 6 | 10 |  |  |  |  |  | |  | 39  (43%) |  |  | 41 / 143  (29%) |
| Bove et al. 2021^13^ | 1993–2004 | 51 | 51 ± 9 | 11 ± 4 | 16 | 38  (75%) |  | 46  (90%) |  | 13  (25%) | | 34  (67%) | 18  (35%) | 36  (71%) | 5  (10%) | 31 / 138  (22%) |
| Total number |  | 351 |  |  |  | 119 / 231 | 47 / 180 | 82 / 107 | 34 / 56 | 118 / 247 | | 118 / 247 | 133 / 351 | 52 / 113 | 49 / 153 | 161 / 573 |
| **Weighted Mean** |  |  | **57.1** | **14.4** | **10.5** |  |  |  |  |  | |  |  |  |  |  |
| **Percentage (95%CI)** |  |  |  |  |  | **52%**  (45–58) | **26%**  (20–33) | **77%**  (68–84) | **61%**  (48–72) | **48%**  (42–54) | | **48%**  (42–54) | **38%**  (33–43) | **46%**  (37–55) | **32%**  (25–40) | **28%**  (25–32) |

In all studies subthalamic deep brain stimulation was used. Abbreviations: DBS = deep brain stimulation; FOG = freezing of gait; FU = follow-up.

**^*^** Cohort characteristics given in medians (range)

^**^ This study also included a control group on conservative treatments with descriptive analysis that did not find differences between groups

**Supplementary Table 3: Long-term controlled observational studies on mortality in DBS treated PD patients**

| **Study** | **n** | **Year of DBS / baseline** | **Cohort characteristics** | | | | **Mortality** | **Notes**  (weaknesses / strengths) |
| --- | --- | --- | --- | --- | --- | --- | --- | --- |
|  |  |  | **Age at**  **onset (years)** | **Age at DBS / baseline**  **(years)** | **Disease duration**  **(years)** | **FU duration** |  |  |
| Schüpbach *et al.*, 2007 ^14^ | 118  STN-DBS | 1996–2004 | 44  (13–61) | 57  (27–75) | – | 41 (7–101) months | 9%  **HR 1.2 (0.7–2.1)** | No details given for controls (historic cohort), short FU, no details of comorbidity in DBS cases |
|  | 39 controls | – | – | – | – | – | – |  |
| Lilleeng *et al.*, 2014 ^15^ | 54  STN-DBS | 2001–2007 | – | 64 ± 6 | – | 8 years | **HR: 1.76 (0.91–3.40)** | No details regarding disease duration, age at onset, or comorbidities. Inclusion bias (only controls with 4 years FU), historic control cohort. |
|  | 54  controls | 1993 | – | 64 ± 6 | – | 18 years | – |  |
| Ngoga *et al.*, 2014 ^16^ | 106  STN-DBS | 2002–2012 | – | 60  (53–63) | 11  (9–13) | < 10 years | 17%  **HR: 0.29 (0.13–0.64)** | No details regarding comorbidity, baseline UPDRS score, and cognition  Controls from the same centre, who were eligible for DBS but choose not to undergo surgery |
|  | 41  controls |  | – | 61  (57–66) | 10  (9–14) |  | 41.5% |  |
| Weaver *et al.*, 2017 ^17^ | 611 DBS | 2006 – 2014 | – | 69 ± 7 | – | 6.3 years | 27%  **HR: 0.69 (0.57-0.85)**  Gain: 7.6 months | Data from medical claims files (no actual study visits), no information on disease severity at baseline, age at onset or disease duration  Large, multicentre cohort, propensity score matching for age, race, Charlson comorbidity index score, depression |
|  | 611  Controls |  | – | 69 ± 9 | – | 5.7 years | 35% |  |
| Mahlknecht *et al.*, 2020 ^18^ | 74 STN-DBS | 1999-2010 | 49  (41–55) | 63  (57–69) | 12 (9–16) | 13 (11–17) years | 36%  **HR: 0.75 (0.43–1.33)** | Low sample size  Detailed baseline data for both groups, controlling for confounders |
|  | 61 controls | 2004 | 51  (46–57) | 63  (55–70) | 8 (5–15) | 15 (14–15) years | 48% |  |

Abbreviations: DBS = Deep Brain Stimulation, FU = Follow Up, HR = Hazard Ratio, STN = Subthalamic nucleus

**References to appendix**

1 Castrioto A, Lozano AM, Poon Y-Y, Lang AE, Fallis M, Moro E. Ten-year outcome of subthalamic stimulation in Parkinson disease: a blinded evaluation. Arch Neurol. 2011;68:1550–6.

2 Zibetti M, Merola A, Rizzi L, et al. Beyond nine years of continuous subthalamic nucleus deep brain stimulation in Parkinson’s disease. Mov Disord. 2011;26:2327–34.

3 Merola A, Romagnolo A, Bernardini A, et al. Earlier versus later subthalamic deep brain stimulation in Parkinson’s disease. Parkinsonism Relat Disord. 2015;21:972–5.

4 Lau B, Meier N, Serra G, et al. Axial symptoms predict mortality in patients with Parkinson disease and subthalamic stimulation. Neurology. 2019;92:e2559–70.

5 Volonté MA, Clarizio G, Galantucci S, et al. Long term follow-up in advanced Parkinson’s disease treated with DBS of the subthalamic nucleus. J Neurol. 2021;268:2821–2830.

6 Fasano A, Romito LM, Daniele A, et al. Motor and cognitive outcome in patients with Parkinson’s disease 8 years after subthalamic implants. Brain. 2010;133:2664–76.

7 Merola A, Zibetti M, Angrisano S, et al. Parkinson’s disease progression at 30 years: a study of subthalamic deep brain-stimulated patients. Brain. 2011;134:2074–84.

8 Rizzone MG, Fasano a., Daniele a., et al. Long-term outcome of subthalamic nucleus DBS in Parkinson’s disease: From the advanced phase towards the late stage of the disease? Park Relat Disord. 2014;20:376–81.

9 Bang Henriksen M, Johnsen EL, Sunde N, Vase A, Gjelstrup MC, Østergaard K. Surviving 10 years with deep brain stimulation for Parkinson’s disease - a follow-up of 79 patients. Eur J Neurol. 2016;23:53–61.

10 Constantinescu R, Eriksson B, Jansson Y, et al. Key clinical milestones 15 years and onwards after DBS-STN surgery-A retrospective analysis of patients that underwent surgery between 1993 and 2001. Clin Neurol Neurosurg. 2017;154:43–8.

11 Lilleeng B, Gjerstad M, Baardsen R, Dalen I, Larsen JP. The long-term development of non-motor problems after STN-DBS. Acta Neurol Scand. 2015;132:251–8.

12 Lilleeng B, Gjerstad M, Baardsen R, Dalen I, Larsen JP. Motor symptoms after deep brain stimulation of the subthalamic nucleus. Acta Neurol Scand. 2015;131:298–304.

13 Bove F, Mulas D, Cavallieri F, et al. Long-term Outcomes (15 Years) After Subthalamic Nucleus Deep Brain Stimulation in Patients With Parkinson Disease. Neurology. 2021; Epub 2021 Jun 2.:10.1212/WNL.0000000000012246.

14 Schüpbach MWM, Welter ML, Bonnet AM, et al. Mortality in patients with Parkinson’s disease treated by stimulation of the subthalamic nucleus. Mov Disord. 2007;22:257–61.

15 Lilleeng B, Brønnick K, Toft M, Dietrichs E, Larsen JP. Progression and survival in Parkinson’s disease with subthalamic nucleus stimulation. Acta Neurol Scand. 2014;130:292–8.

16 Ngoga D, Mitchell R, Kausar J, Hodson J, Harries A, Pall H. Deep brain stimulation improves survival in severe Parkinson’s disease. J Neurol Neurosurg Psychiatry. 2014;85:17–22.

17 Weaver FM, Stroupe KT, Smith B, et al. Survival in patients with Parkinson’s disease after deep brain stimulation or medical management. Mov Disord. 2017;32:1756–63.

18 Mahlknecht P, Peball M, Mair K, et al. Has Deep Brain Stimulation Changed the Very Long-Term Outcome of Parkinson’s Disease? A Controlled Longitudinal Study. Mov Disord Clin Pract. 2020;7:782–7.
